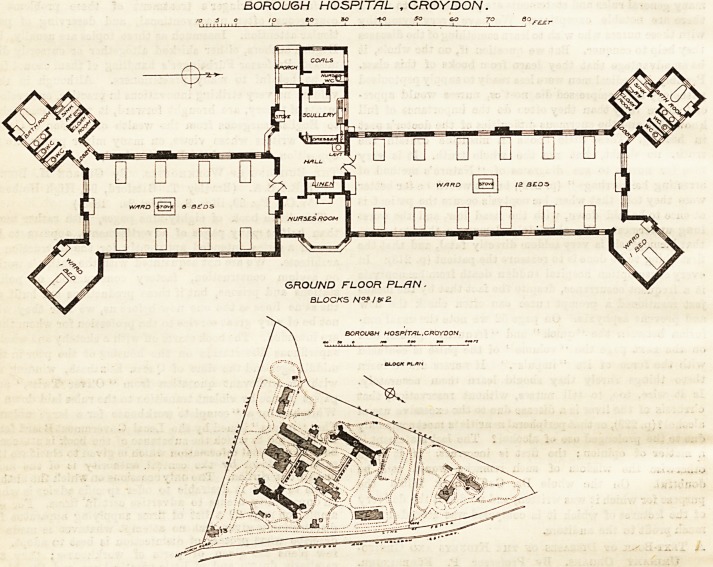# Hospital Construction

**Published:** 1898-08-13

**Authors:** 


### The Institutional Workshop.

### HOSPITAL CONSTRUCTION.

### THE BOROUGH HOSPITAL, CROYDON.

The buildings of the Borough Hospital, Croydon, were described in The Hospital for June 13th, 1896, and are further referred to in "Hospitals and Charities" for 1897, p. 80, so that a general description is unnecessary. The block plan now published shows the new wards in black, the older buildings, some of which are temporary, being hatched to distinguish them. The large-scale plan shows one of the new blocks, accommodating 22 beds, 12 for women and children, eight for men and boys, and two for special cases. The arrangement for the last-named is unusual, the special ward projecting from the angle, and corresponding with the lavatory block. The floor space is 162 ft. per bed, the cubic space over 2,000 ft., and the arrangements generally seem suitable and satisfactory. The hall, however, would appear from the plan to be deficient in light and ventilation, and the lavatory basin placed in it can have neither, unless from a skylight. The buildings have been designed by the Borough Engineer (Mr. Walker), assisted by Mr. Hazlehurst.

BOROUGH HOSPITAL, CROYDON.

GROUND FLOOR PLAN.

BLOCKS Nos 1 & 2

## Figures and Tables

**Figure f1:**